# Multiple Myeloma and the Kidney

**DOI:** 10.1097/HS9.0000000000000556

**Published:** 2021-04-16

**Authors:** Giovanni Cancarini, Vincenzo Terlizzi, Letizia Zeni

**Affiliations:** Nephrology Operative Unit, ASST Spedali Civili Brescia, Brescia, Italy

The recent guidelines by the European Hematology Association (EHA) and European Society for Medical Oncology (ESMO)^1^ address the complexity of the treatment in multiple myeloma (MM). The section on supportive care for renal impairment in the supplementary materials suggests the preferred drugs or the change in their doses according to renal function.

MM can affect the kidney function in several ways, either as monoclonal gammopathy of renal significance (MGRS) or as acute kidney injury (AKI) as in light chain cast nephropathy (LCCN). Surprisingly, renal complications in MM are not mentioned throughout the paper.

A recent work by the International Kidney and Monoclonal Gammopathy Research Group^2^ was focused on treatment of AKI in symptomatic MM and suggested some therapeutic strategies to improve AKI-associated outcomes. In patients with very low glomerular filtration rate (GFR), besides chemotherapy and use of high-permeability dialysis filters (HCO), the abovementioned guidelines suggest hydration and urine alkalinization with the aim to increase both tubular flow and urine pH to reduce intraluminal precipitation of light chains (LCs). Since loop diuretics increase sodium concentration in urine so favoring the precipitation of LCs, the authors suggest that their use should be limited to cases of severe fluid overload only. Another way to increase tubular flow with low effect on intratubular sodium concentration could be intravenously mannitol, which causes an osmotic diuresis thus reducing the risk of cast formation; the use of mannitol in LCCN has recently been supported by a pilot study.^3^ Moreover, it may be the only possibility to remove LCs in countries where HCO dialysis filters are not available due to their high cost

Additionally, if prevention of cast nephropathy is widely considered crucial in the management of MM, the guidelines should emphasize the need of accurate information for all patients with MM on the importance of maintaining high-urine output with neutral pH, avoiding nephrotoxic drugs (FANS, ACE-inhibitors, etc), and having prompt referral to physicians when possible causes of dehydration (vomiting or diarrhea) occur.

## Disclosures

The authors have no conflicts of interest to disclose.
